# Benefits of omega-3 fatty acid against bone changes in salt-loaded rats: possible role of kidney

**DOI:** 10.1002/phy2.106

**Published:** 2013-09-23

**Authors:** Mona A Ahmed, Abeer A Abd EL Samad

**Affiliations:** 1 Department of Physiology, Faculty of Medicine, Ain Shams UniversityCairo, Egypt; 2 Department of Histology, Faculty of Medicine, Ain Shams UniversityCairo, Egypt

**Keywords:** Bone, omega-3, renal function, salt intake

## Abstract

There is evidence that dietary fats are important components contributing in bone health and that bone mineral density is inversely related to sodium intake. Salt loading is also known to impose negative effects on renal function. The present study aimed to determine the effect of the polyunsaturated fatty acid omega-3 on bone changes imposed by salt loading, highlighting the role of kidney as a potential mechanism involved in this effect. Male Wistar rats were divided into three groups: control group, salt-loaded group consuming 2% NaCl solution as drinking water for 8 weeks, and omega-3-treated salt-loaded group receiving 1 g/kg/day omega-3 by gavage with consumption of 2% NaCl solution for 8 weeks. Systolic blood pressure (SBP), diastolic blood pressure (DBP), mean arterial pressure (MAP), and heart rate (HR) were recorded. Plasma levels of sodium, potassium, calcium, inorganic phosphorus (Pi), alkaline phosphatase (ALP), creatinine, urea, 1,25-dihydroxyvitamin D [1,25(OH)_2_D_3_], and transforming growth factor-beta1 (TGF-β1) were measured. The right tibia and kidney were removed for histologic examination and renal immunohistochemical analysis for endothelial nitric oxide synthase (eNOS) was performed. The results revealed that omega-3 reduced SBP, DBP, and MAP and plasma levels of sodium, potassium, Pi, creatinine, urea, and TGF-β1, but increased plasma levels of calcium, ALP, and 1,25(OH)_2_D_3_ as well as renal eNOS. Omega-3 increased cortical and trabecular bone thickness, decreased osteoclast number, and increased newly formed osteoid bone. Renal morphology was found preserved. In conclusion, omega-3 prevents the disturbed bone status imposed by salt loading. This osteoprotective effect is possibly mediated by attenuation of alterations in Ca^2+^, Pi, and ALP, and improvement of renal function and arterial blood pressure.

## Introduction

The acquisition and maintenance of bone mass and strength are influenced by environmental factors, including physical activity and nutrition (Massey and Whiting 1996). Nutrition is important to bone health, and a number of minerals and vitamins have been identified as playing a potential role in the prevention of bone diseases, particularly osteoporosis (Massey and Whiting 1996). Evidence indicates that dietary fats can influence bone health (Tartibian et al. 2010), in particular the omega-3 (n-3) polyunsaturated fatty acids (PUFAs), as they have been shown to inhibit osteoclast activity and enhance osteoblast activity (Watkins et al. 2003). Eicosapentaenoic acid (EPA) supplementation was found to increase bone mineral density in postmenopausal women (Terano 2001). Beneficial effects of n-3 PUFAs on markers of bone resorption and formation in animal (Shen et al. 2006) and human (Griel et al. 2007) studies have, also, been observed.

On the other hand, a number of studies suggested a detrimental effect of dietary salt on bone. Devine et al. (1995) showed that change in bone mineral density was inversely related to sodium intake and that both dietary calcium and urinary sodium excretion were significant determinants of the change in bone mass. High-sodium diet was found to increase urinary calcium excretion and cause loss of bone calcium (Chan and Swaminathan 1998), while reducing sodium intake complemented the beneficial skeletal effects of the Dietary Approaches to Stop Hypertension diet (Lin et al. 2003). Furthermore, an epidemiological study of men and women has shown that salt intake is associated with markers of bone resorption and appears likely to be a risk factor for osteoporosis (Jones et al. 1997). Similar effect of sodium loading has been demonstrated in animal model (Gold and Gouldin 1995).

In addition, studies in humans and animals have demonstrated that high-salt intake induces extensive target organ damage, with the kidneys inevitably involved (Yu et al. 1988; Mimran and du Cailar 2008).

It was, therefore, worthwhile to clarify the possible beneficial effect of omega-3 fatty acid supplementation on altered bone status in rats maintained on high-salt intake, and to assess renal function and structure in a trial to shed light on a possible causal role of the kidney in the assumed bone changes.

## Materials and Methods

### Experimental animals

This work was performed on 25 adult male Wistar rats, weighing 200–280 g, purchased from the experimental animal farm in El Giza, and maintained in the Physiology Department Animal House under standard conditions of boarding. All rats were maintained on ad libitum standard diet composed of carbohydrates (50–51%), proteins (12–13%), and fats (4–5%) according to the normal dietary requirements (Cuthbertson 1957; Hallfrisch et al. 1979). The Faculty of Medicine Ain Shams University Research Ethics Committee (FMASU REC), Cairo, Egypt approved all experimental procedures.

### Experimental protocol

Animals were divided into the following groups:

- **Control group** (*n* = 8) consisting of rats consuming tap water.
- **Salt-loaded group** (*n* = 8) consisting of rats consuming 2% NaCl solution as drinking water (Cruz et al. 2011) for the 8 weeks of the experiment.
- **Omega-3 fatty acid-treated salt-loaded group** (*n* = 9) consisting of rats receiving omega-3 fatty acid (SEDICO, 6 October City, Egypt) daily at a dose of 1 g/kg b.w. (Fernandez et al. 2004) orally by gavage, with consumption of 2% NaCl solution as drinking water for 8 weeks. Administration of the omega-3 fatty acids by gavage did not interfere with consumption of saline or the diet.

## Experimental procedures

Body weight, tail systolic blood pressure (SBP), diastolic blood pressure (DBP), and mean arterial pressure (MAP) as well as heart rate (HR) of rats in the three groups were measured at the beginning of the study (basal values) and at the end of the study period (final values). Tail SBP, DBP, MAP, and HR were measured using rat tail noninvasive blood pressure system (NIBA 200A; Biopac Systems, Inc., Goleta, CA).

On the day of experiment, overnight fasted rats, except with free access to water, were weighed and anesthetized i.p. with 40 mg/kg b.w. thiopental sodium (EIPICO, 10th of Ramadan City, Egypt). A midline abdominal incision was made, and the abdominal aorta was exposed and cannulated. Blood samples were collected in heparinized tubes, and centrifuged at 3000 rpm for 15 min. to separate plasma. The obtained plasma was subjected to measurement of sodium, potassium, calcium, inorganic phosphorus (Pi), alkaline phosphatase (ALP) enzyme activity, creatinine, urea, 1,25-dihydroxyvitamin D [1,25 (OH)_2_D_3_], and transforming growth factor-beta1 (TGF-β1). The right tibia and the right kidney were dissected out, fixed in 10% neutral buffered formaldehyde, and subjected to histological studies.

### Biochemical assays

Plasma levels of Na^+^, K^+^, Ca^2+^, Pi, ALP activity, creatinine, and urea were measured by colorimetric methods using kits supplied by Bio-diagnostic, Egypt. Plasma 1,25-dihydroxyvitamin D was measured using 1,25-dihydroxyvitamin D ^125^I RIA (DiaSorin Inc., Stillwater, MN) and plasma total TGF-β1was determined by TGF-β1 ELISA kit (Biosource International, Inc., Camarillo, CA). All measurements were performed according to the manufacturer's instructions.

### Bone and kidney processing for histological examination

#### Bone specimens

The right tibia specimens were dissected out and fixed in 10% neutral buffered formaldehyde for 2 days. Then, they were decalcified using the chelating agent, formalin-EDTA (ethylene-diamine-tetra-acetic acid, disodium salt) for 4 weeks. The solution was changed every day. The specimens were then processed for light microscopic study to get paraffin sections of 5-μm thickness. They were stained with Hematoxylin and Eosin (H&E) and Masson's trichrome to examine the proximal metaphysises of tibia (Bancroft and Gamble 2008).

#### Kidney specimens

The right kidney specimens were dissected, cut longitudinally, fixed in 10% neutral buffered formaldehyde, and then processed for light microscopic study to get paraffin sections of 5-μm thickness. Some sections were stained with H&E for morphological studies, and some were subjected to immunohistochemical technique for endothelial nitric oxide synthase (eNOS) (Bancroft and Gamble 2008).

For the immunohistochemistry study, serial paraffin sections were deparaffinized and dehydrated, including the positive control sections. The endogenous peroxidase activity was blocked with 0.05% hydrogen peroxide in absolute alcohol for 5 min. The slides were washed for 5 min in phosphate-buffered saline (PBS) at pH = 7.4. To unmask the antigenic sites, sections were put into 10 mmol/L citrate buffer (pH = 6) in the microwave for 10 min followed by cooling at room temperature for 20 min. The slides were incubated in 1% bovine serum albumin dissolved in PBS for 30 min at 37°C in order to prevent the nonspecific background staining. The monoclonal primary antibody with dilution of 1:50 of eNOS (Lab Vision, Fermont, CA) was applied to sections except for negative control. Then, they were incubated for 60 min at room temperature. The slides were rinsed with PBS, then incubated for 60 min with anti-mouse immunoglobulins (secondary antibody) conjugated to peroxidase labeled dextran polymer (DAKO, Glostrup, Denmark). In order to detect the reaction, the slides were incubated in 3,3-diaminobenzidine (DAB) for 15 min. The slides were counterstained by Hematoxylin, then dehydrated, cleared, and mounted by a mixture of distyrene, a plasticizer and xylene (DPX).

### Scoring of kidney

Kidney changes as glomerular hypercellularity, renal tubular affection (dilatation of lumen and/or degenerated cells), lesions of renal cortical vessels (thickening of media), and interstitial cellular infiltration were estimated in H&E-stained sections of all animals using Zeiss microscope in Histology department, Faculty of Medicine, Ain Shams University. They were scored from 0 to 3 as: score 0 = no abnormalities, score 1 = changes affecting less than one-third of the specimen, score 2 = changes affecting one to two-thirds of the specimen, and score 3 = changes affecting more than two-thirds of the specimen (Lloberas et al. 2001).

### Morphometric study

The mean trabecular bone thickness (TBT) in μm, the mean cortical bone thickness (CBT) in μm, the mean of osteoclast number/ high-power field (HPF), and the area percent (%) of eNOS immunoreactivity in renal cortex were measured in five fields from three serial sections of each animal, using Digimizer version 4.1.1.0, copyright © 2005–2011 MedCalc software (bvba, Mariakerke, Belgium). The CBT was measured by drawing a vertical line from just beneath the periosteum to the endosteum, and the TBT was measured in cancellous bone of metaphysises at their midpoint away from their branching areas.

### Statistical analysis

The statistical data and statistical significance were performed using SPSS statistical package (SPSS Inc., Chicago, IL) version 16.0.1. The statistical significance was determined by one-way analyses of variance (ANOVA) for differences among means of different groups followed by least significant difference test (LSD) to find intergroupal significance. Chi-squared and Mann–Whitney *U*-tests were used to compare renal histological nonparametric data. Pearson's correlation was used to assess relationships between variables. A probability of *P* < 0.05 was considered statistically significant.

## Results

### Body weight

The body weight showed insignificant differences among the control (249.4 ± 4.57 g), salt-loaded (241.6 ± 9.14 g), and omega-3 fatty acid-treated salt-loaded (234.4 ± 5.74 g) groups.

### Arterial blood pressure and HR

Table 1 shows significant increase in final values of SBP, DBP, and MAP of salt-loaded rats as compared to the respective values in the control group and to their respective basal values. Omega-3 treatment decreased SBP, DBP, and MAP. However, HR showed nonsignificant differences among the three groups.

**Table 1:** Changes of systolic blood pressure (SBP), diastolic blood pressure (DBP), mean arterial pressure (MAP), and heart rate (HR) of the different groups

	Control (*n* = 8)	Salt loaded (*n* = 8)	Omega-3 salt loaded (*n* = 9)
SBP, mmHg			
Basal	108 ± 2.60	110 ± 2.95	108 ± 4.93
Final	105 ± 1.46	149^1^,^2^ ± 8.63	112^3^ ± 1.96
DBP, mmHg			
Basal	74 ± 2.09	81 ± 4.70	74 ± 4.16
Final	79 ± 1.13	104^1^,^2^ ± 3.86	90^1^,^2^,^3^ ± 1.56
MAP, mmHg			
Basal	91 ± 2.08	95 ± 3.75	90 ± 4.21
Final	90 ± 1.58	121^1,^^2^ ± 4.96	101^1,^^2^,^3^ ± 1.60
HR, bpm			
Basal	375 ± 22.8	374 ± 20.7	350 ± 12.7
Final	390 ± 16.6	379 ± 21.5	357 ± 12.6

Data expressed as means ± SEM. Number in parenthesis is the number of rats.

1 Significance from respective basal value, calculated by “Student's *t*-test” for paired data at *P* < 0.05.

2 Significance calculated by LSD at *P* < 0.05 from control group.

3 Significance calculated by LSD at *P* < 0.05 from salt-loaded group.

### Biochemical markers

Salt-loaded group demonstrated significant increase in plasma levels of Na^+^, K^+^, Pi, creatinine, urea, and TGF-β1, and significant decrease in plasma levels of Ca^2+^, ALP activity, and 1,25 (OH)_2_D_3_. These changes were alleviated by omega-3 supplementation (Table 2).

**Table 2:** Changes of biochemical markers in plasma of the different groups

	Control (*n* = 8)	Salt loaded (*n* = 8)	Omega-3 salt loaded (*n* = 9)
Na^+^, mmol/L	117.4 ± 3.59	145.1^1^ ± 4.32	129.5^2^ ± 6.44
K^+^, mmol/L	3.10 ± 0.19	3.98^1^ ± 0.17	3.33^2^ ± 0.26
Ca^2+^, mg/dL	8.98 ± 0.01	7.30^1^ ± 0.11	8.53^1^,^2^ ± 0.16
Pi, mg/dL	3.55 ± 0.30	4.81^1^ ± 0.29	3.57^2^ ± 0.12
ALP, IU/L	83.6 ± 8.54	62.2^1^ ± 5.33	82.9^2^ ± 7.02
Creatinine, mg/dL	0.62 ± 0.04	1.12^1^ ± 0.02	0.78^1^,^2^ ± 0.05
Urea, mg/dL	38.6 ± 2.24	64.0^1^ ± 6.93	38.7^2^ ± 1.81
1,25 (OH)_2_D_3_, pg/mL	58.9 ± 3.49	21.0^1^ ± 1.32	34.7^1^,^2^ ± 1.42
TGF-β1, pg/mL	50.8 ± 4.16	227.6^1^ ± 14.2	127.8^1^,^2^ ± 4.02

Data expressed as means ± SEM. Number in parenthesis is the number of rats.

1 Significance calculated by LSD at *P* < 0.05 from control group.

2 Significance calculated by LSD at *P* < 0.05 from salt-loaded group.

The correlations between the different variables are illustrated in Table 3.

**Table 3:** Correlations between the different variables

Variables for correlation	Statistical analysis	

	Correlation coefficient (*r*)	*P*-value
Creatinine		
Ca^2+^	−0.886	<0.001
Pi	0.494	<0.05
1,25(OH)_2_D_3_	−0.734	<0.001
ALP	−0.482	<0.02
Urea		
Ca^2+^	−0.635	<0.005
Pi	0.754	<0.001
1,25(OH)_2_D_3_	−0.495	<0.05
ALP	−0.364	NS
TGF-β1		
Creatinine	0.821	<0.001
Urea	0.593	<0.005
SBP		
Creatinine	0.692	<0.001
Urea	0.739	<0.001

The number of rats studied for each correlation is 25. NS, not significant.

### Bone histological changes

**Figure 1:**
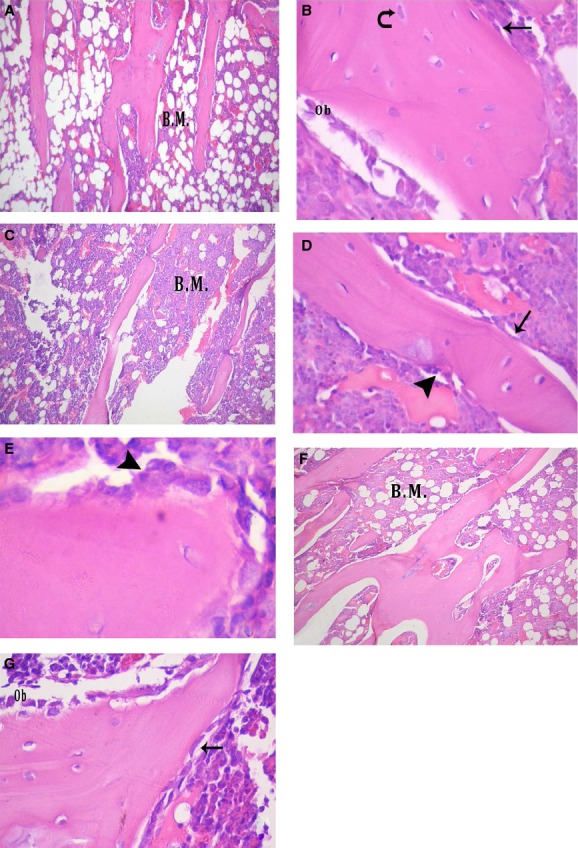
Metaphysis of upper end of tibia of: (A and B) Control group: (A) showing irregular cancellous bone trabeculae with bone marrow (B.M.) tissue in-between (H&E 200 × ). (B) Showing osteocytes (curved arrow), osteoprogenitor cells (↑), and osteoblasts (Ob) in relation to bone trabeculae (H&E 640 × ). (C and E) Salt-loaded group: (C) showing apparent thinning of bone trabeculae and widening of B.M. tissue in-between them (H&E 200 × ), (D) showing eroded surface of the bone trabeculae with osteoclast (▲) and osteoprogenitor cells (↑) lining the endosteum on the other side of the trabeculum (H&E 640 × ), (E) showing osteoclasts (▲) lining the resorption area on bone surface (H&E 1500 × ). (F and G) Omega-3-treated salt-loaded group: (F) showing apparent thickening of bone trabeculae compared to salt-loaded group with B.M. tissue in-between them (H&E 200 × ), (G) showing osteoprogenitor cells (↑) and osteoblasts (Ob) lining endosteum of bone trabeculae (H&E 640 × ).

The examination of H&E sections of control group showed that the metaphysis of upper end of tibia had an outer shell of compact or cortical bone and inner trabeculae of cancellous bone. The irregular cancellous bone trabeculae appeared with osteocytes inside lacunae. Both osteoprogenitor cells and osteoblasts were seen lining the endosteum of the bone trabeculae. Bone marrow spaces and tissue were present in-between these trabeculae (Fig. 1A and B). Salt-loaded group sections revealed apparent thinning of the bone trabeculae and widening of bone marrow tissue in-between them. Many irregular resorption areas on bone surface were seen lined by osteoclasts (Fig. 11C–E), whereas omega-3 salt-loaded group sections showed apparent thickening of bone trabeculae compared to the salt-loaded group with many osteoblasts lining endosteum of bone trabeculae (Fig. 1F and G).

**Figure 2:**
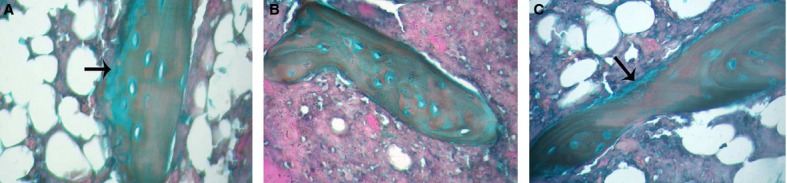
Metaphysis of upper end of tibia of: (A) Control group showing newly formed osteoid bone with light green color (↑). (B) Salt-loaded group showing apparent less newly formed osteoid bone. (C) Omega-3-treated salt-loaded group showing apparent increased newly formed osteoid bone (↑) compared to salt-loaded group (Masson's trichrome 400 × ).

**Figure 3:**
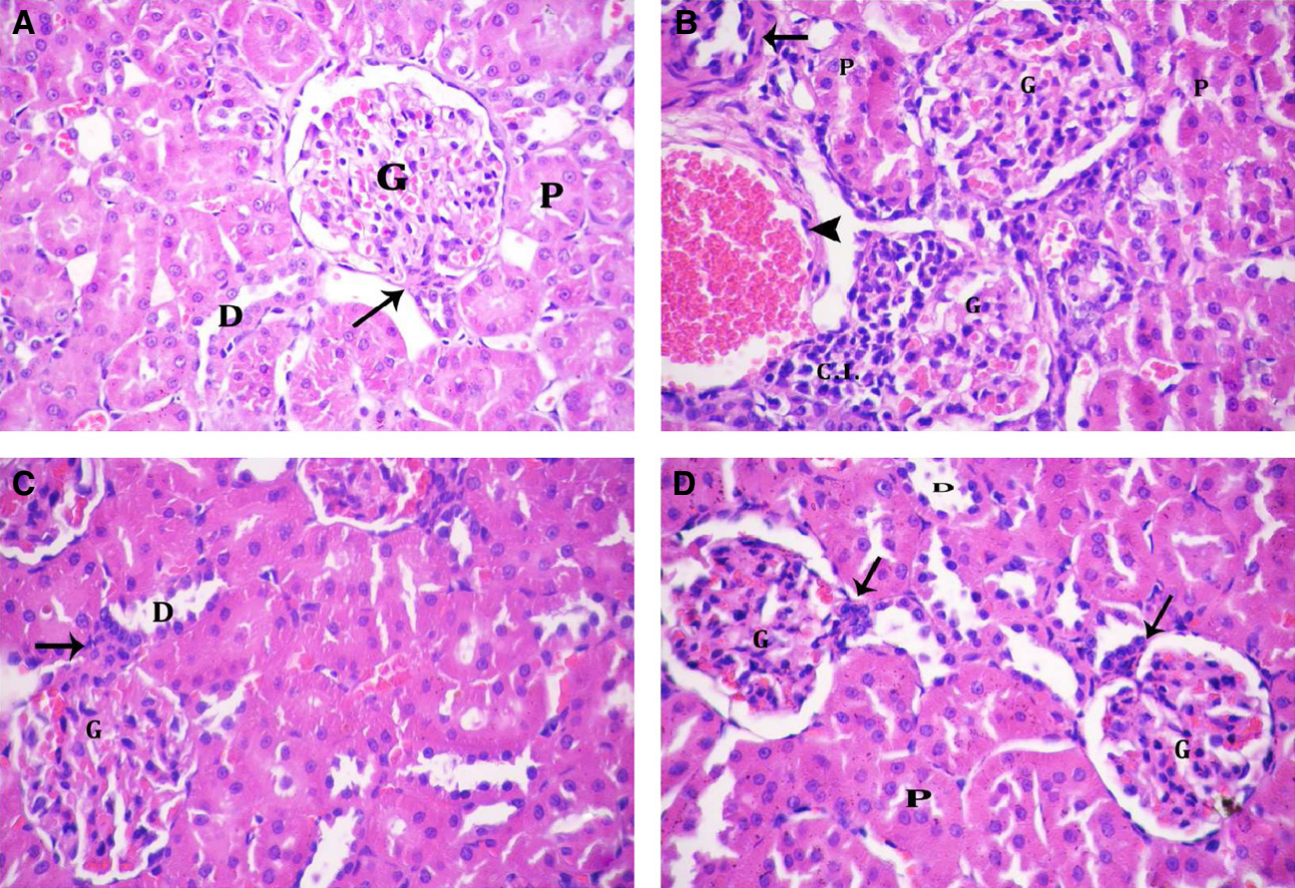
Renal cortex of: (A) Control group showing renal corpuscle with normal glomerulus (G). The juxtaglomerular apparatus (↑) is seen. Notice normal pattern of proximal convoluted (P) and distal convoluted (D) tubules. (B and C) Salt-loaded group: (B) showing most of the corpuscles (G) with high cellularity and obliterated capsular space. Proximal convoluted tubules (P) show destructed epithelial lining. Notice congested blood vessels (▲), thickened arterioles (↑), and cellular infiltration (C.I.). (C) Showing high cellularity of renal corpuscles (G) and extra-mesangial cells (↑) of juxtaglomerular apparatus. Notice destructed epithelial lining of distal convoluted tubules (D). (D) Omega-3-treated salt-loaded group showing preservation of normal pattern of renal corpuscles (G) and juxtaglomerular apparatus (↑). Both proximal (P) and distal (D) convoluted tubules appear nearly normal (H&E 400 × ).

**Figure 4:**
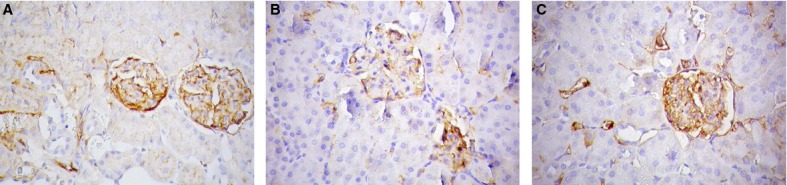
Renal cortex of: (A) Control group showing eNOS-positive immunoreactivity of endothelial cells of both glomeruli and intertubular capillaries. (B) Salt-loaded group showing apparent less intense eNOS immunoreactivity. (C) Omega-3-treated salt-loaded group showing apparent increased eNOS immunoreactivity compared to salt-loaded group (eNOS 400 × ).

Morphometric study revealed a significant decrease in TBT and CBT of salt-loaded group compared to control and a significant increase in TBT and CBT of omega-3 salt-loaded group compared to salt-loaded group. The osteoclast number showed significant increase in salt-loaded group compared to control group and significant decrease in omega-3 salt-loaded group compared to salt-loaded group (Table 4).

**Table 4:** Trabecular bone thickness (TBT), cortical bone thickness (CBT), and number of osteoclasts/HPF of the different groups

	Control	Salt loaded	Omega-3 salt loaded
TBT, μm	89.2 ± 0.73	54.6^1^ ± 2.67	84.4^2^ ± 1.38
CBT, μm	284.7 ± 11.6	238.7^1^ ± 10.6	280.3^2^ ± 4.63
Number of osteoclasts/HPF	0.43 ± 0.08	1.85^1^ ± 0.19	0.63^2^ ± 0.09

Data expressed as means ± SEM.

1 Significance calculated by LSD at *P* < 0.05 from control group.

2 Significance calculated by LSD at *P* < 0.05 from salt-loaded group.

Examination of Masson's trichrome-stained section of control group showed light green color of newly formed osteoid bone in contrast to mature bone with dark greenish color (Fig. 2A). Sections of salt-loaded group revealed apparent less newly formed osteoid bone (Fig. 2B), whereas sections of omega-3-treated salt-loaded group showed apparent increased newly formed osteoid bone compared to salt-loaded group (Fig. 2C).

### Renal histological changes

Examination of H&E sections of renal cortex of control rats showed renal corpuscles with normal glomerulus and capsular space surrounded by Bowman's capsule. The juxtaglomerular apparatus is seen formed of macula densa, juxtaglomerular cells, and extra-mesangial cells. Proximal convoluted tubules appeared lined by tall cuboidal cells with basal round vesicular nuclei and apical brush border surrounding a narrow lumen. Distal convoluted tubules appeared with wider lumen, lined by low cuboidal cells having round central bulging nuclei (Fig. 3A). On the other hand, renal cortex of salt-loaded group showed that renal corpuscles and extra-mesangial cells of juxtaglomerular apparatus appeared with high cellularity. The capsular space appeared obliterated. Most of proximal and distal convoluted tubules had destructed epithelial lining with some darkly stained nuclei. Congested blood vessels and thickened arterioles were detected with interstitial cellular infiltration (Fig. 3B and C). However, in sections of omega-3-treated salt-loaded group, there was preservation of normal pattern of most of renal corpuscles and juxtaglomerular apparatus. Both proximal and distal convoluted tubules appeared nearly normal (Fig. 3D).

The renal grading and scoring revealed a significant increase in glomerular hypercellularity, renal tubular affection, vascular lesions, and interstitial cellular infiltration in salt-loaded group compared to control group and a significant decrease in these parameters in omega-3 salt-loaded group compared to salt-loaded group (Table 5).

**Table 5:** Kidney scoring parameters and area percent (%) of endothelial nitric oxide synthase (eNOS) immunoreactivity of the different groups

	Control	Salt loaded	Omega-3 salt loaded
Glomerular hypercellularity	0.00 (0.00–0.00)	3.00^1^ (3.00–3.00)	0.50^2^ (0.00–1.00)
Renal tubular affection	0.00 (0.00–0.00)	3.00^1^ (2.00–3.00)	1.50^1^,^2^ (1.00–2.00)
Lesions of renal cortical vessels	0.00 (0.00–0.00)	3.00^1^ (2.25–3.00)	2.00^1^,^2^ (1.25–2.75)
Interstitial cellular infiltration of renal cortex	0.00 (0.00–0.00)	2.00^1^ (2.00–2.00)	1.00^1,^^2^ (0.25–1.00)
Area% of eNOS immunoreactivity	11.35 (8.55–12.35)	5.05^1^ (4.83–5.18)	9.50^2^ (8.45–10.5)

Data expressed as median (interquartile range). The nonparametric Chi-squared test and Mann–Whitney *U*-test evaluated kidney scoring parameters and area% of eNOS immunoreactivity, respectively.

1 Significance calculated at *P* < 0.05 from control group.

2 Significance calculated at *P* < 0.05 from salt-loaded group.

The immunohistochemically stained sections with eNOS antibody of control group showed positive immunoreactivity of endothelial cells of both glomeruli and intertubular capillaries (Fig. 4A). The sections of salt-loaded group showed apparent less intense immunoreactivity of endothelial cells of both glomeruli and intertubular capillaries for eNOS (Fig. 4B), whereas those of omega-3-treated salt-loaded group revealed apparent increased immunoreactivity of endothelial cells of both glomeruli and intertubular capillaries for eNOS compared to salted-loaded group (Fig. 4C).

Morphometric study showed that the area percent (%) of eNOS immunoreactivity was significantly decreased in salt-loaded group compared to control group and significantly increased in omega-3 salt-loaded group compared to salt-loaded group (Table 5).

## Discussion

The data obtained in this study revealed reduction in plasma Ca^2+^, 1,25(OH)_2_D_3_ and ALP activity, the bone formation marker, and elevation of plasma Pi in rats exposed to high-salt intake. Such changes were accompanied by and correlated with the altered bone integrity, denoted by enhanced bone resorption, and decreased bone formation, that is observed histologically in the form of thinning of trabecular and cortical bones, many irregular resorption areas on bone surface together with increased number of osteoclasts and decreased new bone formation. These findings are in agreement with previous studies showing deleterious effect of high salt on bone (Devine et al. 1995; Gold and Gouldin 1995; Jones et al. 1997; Chan and Swaminathan 1998; Lin et al. 2003). Also, high-salt intake increased SBP, DBP, and MAP, a result that is in line with earlier reports (Newaz et al. 2005; Vargas et al. 2006; Cruz et al. 2011).

Moreover, high-salt intake was associated with increased plasma levels of creatinine, urea, and K^+^, pointing to deranged kidney function, which could be explained by the disrupted kidney structure, seen by histological examination as glomerular hypercellularity, proximal and distal tubular affection, congested renal cortical vessels, and interstitial cellular infiltration. In agreement, previous studies showed that excessive salt intake exerts severe detrimental effects on renal structure and function (Dahl et al. 1963; Barsanti et al. 1971). Some investigators suggested that the increased arterial pressure associated with excess salt intake was partially responsible for inflicting renal damage (Yu et al. 1988; Matavelli et al. 2007), a view which is supported in the current work by the positive correlations of blood pressure with the renal function markers (creatinine and urea).

Furthermore, salt-loaded rats exhibited decreased renal eNOS, suggestive of reduced NO bioavailability, together with high plasma level of TGF-β1, a fibrogenic growth factor, both possibly contributing in pathogenesis of the salt-induced hypertension as well as in the apparent renal tissue and renal vascular lesions detected by histological examination. High salt was found to decrease expression of cortical and glomerular eNOS and to increase cortical expression of TGF-β1 (Fiore et al. 2011) that hastens the progression of kidney disease (Nakayama et al. 2009) and correlates with vascular injury severity (Hohenstein et al. 2008). Chronic NOS inhibition leads to sodium retention, volume expansion, and volume-dependent hypertension as NO exerts a direct tubular effect to inhibit sodium reabsorption and promotes the pressure natriuresis (Stoos et al. 1992; Majid et al. 1993). Also, it was found that in normotensive rats, excess salt intake rapidly increased endothelial production of active TGF-β (Ying and Sanders 1999) that promotes vascular stiffness, vasoconstriction, and hypertension (Kanbay et al. 2011).

The renal function impairment demonstrated herein is suggested to be involved in the salt-mediated bone changes. It has been proposed that defective kidney function decreases phosphate excretion with resultant hyperphosphatemia, which could explain the high Pi level observed in the high-salt group. Increased serum phosphate inhibits 1α hydroxylase and produces fall in 1,25(OH)_2_D_3_ resulting in diminished intestinal Ca^2+^ absorption and reduced Ca^2+^ level in blood, observed in the present study, and its availability to bones. The decreased Ca^2+^ level could, also, be directly attributed to diminished renal Ca^2+^ reabsorption resulting from renal tubular damage, as shown by the renal histologic picture. The high Pi together with low Ca^2+^ and 1,25(OH)_2_D_3_ levels are known to stimulate secretion of parathyroid hormone (PTH), with secondary hyperparathyroidism; the accumulating PTH stimulating osteoclastic activity (Brenner and Lazarus 1991; Bover et al. 1999).

Furthermore, it has been reported that high-dietary sodium intake decreases renal Ca^2+^ reabsorption, which in turn leads to a greater urinary calcium excretion and loss of bone Ca^2+^ (Chan and Swaminathan 1998; Sarić and Piasek 2005), thus providing further explanation to salt-mediated bone alterations.

Administration of omega-3 fatty acid was able to prevent bone alterations induced by high-salt intake resulting in increased bone mass and formation and decreased bone resorption and loss, as evidenced by the histological findings of increased TBT and CBT, decreased number of osteoclasts, many osteoblasts, and increased newly formed osteoid bone. Meanwhile, omega-3 increased plasma ALP, Ca^2+^, and 1,25(OH)_2_D_3_ levels and decreased the Pi level, thereby promoting increased bone mass. This confirms the positive influence of omega-3 on bone formation. In concordance with the present results, increased calcium absorption after dietary intake of n-3 fatty acids has been reported in humans (Heaney et al. 2005) and in young male rats (Claassen et al. 1995). Also, increased ALP has been reported in osteoblast-like cells treated with n-3 fatty acids and in young as well as middle-aged Sprague–Dawley rats treated with dietary n-3 fatty acids (Watkins et al. 2003; Shen et al. 2006). The n-3 PUFAs have been shown to inhibit the activity of osteoclasts and to enhance the activity of osteoblasts, thereby inhibiting bone resorption and promoting bone formation (Sun et al. 2003; Coetzee et al. 2007; Tartibian et al. 2010). Long-term supplementation of n-3 fatty acids in the form of fish oil was found to increase bone mineral content, mechanical properties, and histological properties of Japanese quail (Liu et al. 2003).

Also, omega-3 fatty acid supplementation to salt-loaded rats prevented the renal dysfunction as evidenced by decreased plasma creatinine, urea, and K^+^ levels and, as well, preserved renal morphology. Omega-3-mediated prevention of renal changes could be attributed to omega-3-induced antihypertensive effect together with lowering of TGF-β1. Increasing evidence shows that TGF-β1 is a key mediator in the pathogenesis of renal diseases in both experimental and human studies (Nakayama et al. 2009; Fiore et al. 2011). The significant positive correlations found between plasma TGF-β1 and plasma creatinine and urea suggest that salt-induced increase in plasma TGF-β1 is involved in impairment of renal function. Similar to the present results, Barcelli et al. (1986) reported favorable effects of fish oil on progression of renal disease and kidney histology in rats with renal mass reduction. EPA administration was shown to attenuate oxidative stress, lower TGF-β abundance, and ameliorate renal injury in mice with type 2 diabetic nephropathy (Zhang et al. 2006).

Omega-3-induced prevention of excess salt-related renal functional and structural derangements could contribute in its bone-preserving effect, through restoration of renal Ca^2+^ and Pi handling, increasing plasma levels of Ca^2+^ and decreasing Pi plasma level. This notion is supported by renal function markers (creatinine and urea) negative correlations with calcium, 1,25(OH)_2_D_3_, and ALP plasma levels and positive relations with plasma Pi.

The suppression of the hypertension following treatment with omega-3 could provide an additional mechanism for the beneficial effect of omega-3 on bone and could be explained by omega-3-induced increase in eNOS and NO bioavailability and decrease in Na^+^ and TGF-β1, findings which are in consistence with previous studies (Medeiros et al. 2005; Fischer et al. 2008; An et al. 2009; Chen et al. 2011). In agreement, former studies showed that omega-3 PUFAs significantly decreased systemic arterial blood pressure in hypertension models of transgenic rats expressing the human renin and angiotensinogen genes (Fischer et al. 2008) and diabetic spontaneously hypertensive rats (Medeiros et al. 2005). Chen et al. (Chen et al. 2011) showed that omega-3 PUFAs, EPA and docosahexaenoic acid, increased phosphorylated eNOS and, as well, the eNOS levels and NO production in cardiac fibroblasts. An et al. (2009) showed that omega-3 supplementation significantly attenuated upregulations of TGF-β1 in the remnant kidneys in rats with chronic renal failure.

It could, thus, be concluded that omega-3 supplementation prevented the disturbed bone status imposed by salt loading. This osteoprotective effect might be possibly mediated by attenuation of alterations in Ca^2+^, Pi, and ALP and improvement of renal function and arterial blood pressure. Thus, omega-3 is a potentially useful natural agent in the preservation of bone integrity in high-salt consumers.

### Study limitations

Several limitations should be mentioned for the present study. First, the exact effect of omega-3 fatty acids alone on the different studied parameters was not determined as a control group ingesting omega-3 fatty acids alone was not included in the study. Second, the used salt load induced intrinsic renal damage which could have interfered with the bone changes; it remains questionable if the use of a moderate salt load might have enabled to specify the effect of salt alone on bone histology. Third, although the decalcified specimens used in the histology provided obvious and significant changes that were very satisfactory, the question arises if the use of calcified specimens could have allowed us to comment on more range of bone changes. Therefore, future studies are recommended in order to provide answers to these inquires.

## References

[b1] An WS, Kim HJ, Cho KH, Vaziri ND (2009). Omega-3 fatty acid supplementation attenuates oxidative stress, inflammation, and tubulointerstitial fibrosis in the remnant kidney. Am. J. Physiol. Renal Physiol.

[b2] Bancroft JB, Gamble M (2008). Theory and practice of histological techniques.

[b3] Barcelli UO, Miyata J, Ito Y, Gallon L, Laskarzewski P, Weiss M (1986). Beneficial effects of polyunsaturated fatty acids in partially nephrectomized rats. Prostaglandins.

[b4] Barsanti JA, Pillsbury HR, Freis ED (1971). Enhanced salt toxicity in the spontaneously hypertensive rat. Proc. Soc. Exp. Biol. Med.

[b5] Bover J, Jara A, Trinidad P, Rodriguez M, Felsenfeld AJ (1999). Dynamics of skeletal resistance to parathyroid hormone in the rat: effect of renal failure and dietary phosphorus. Bone.

[b6] Brenner BM, Lazarus JM, Wilson JD, Braunwald E, Isselbacher KJ, Petersdorf RB, Martin JB, Fauci AS (1991). Chronic renal failure: pathophysiologic and clinical considerations. Harrison's principles of internal medicine.

[b7] Chan EL, Swaminathan R (1998). Calcium metabolism and bone calcium content in normal and oophorectomized rats consuming various levels of saline for 12 months. J. Nutr.

[b8] Chen J, Shearer GC, Chen Q, Healy CL, Beyer AJ, Nareddy VB (2011). Omega-3 fatty acids prevent pressure overload-induced cardiac fibrosis through activation of cyclic GMP/protein kinase G signaling in cardiac fibroblasts. Circulation.

[b9] Claassen N, Coetzer H, Steinmann CM, Kruger MC (1995). The effect of different n-6/n-3 essential fatty acid ratios on calcium balance and bone in rats. Prostaglandins Leukot. Essent. Fatty Acids.

[b10] Coetzee M, Haag M, Kruger MC (2007). Effects of arachidonic acid, docosahexaenoic acid, prostaglandin E(2) and parathyroid hormone on osteoprotegerin and RANKL secretion by MC3T3-E1 osteoblast-like cells. J. Nutr. Biochem.

[b11] Cruz A, Rodríguez-Gómez I, Pérez-Abud R, Vargas MA, Wangensteen R, Quesada A (2011). Effects of clofibrate on salt loading-induced hypertension in rats. J. Biomed. Biotechnol.

[b12] Cuthbertson WF (1957). Nutrient requirements of rats and mice. Proc. Nutr. Soc.

[b13] Dahl LK, Heine M, Tassinari L (1963). Effects of chronic excess salt ingestion. Role of genetic factors in both doca-salt and renal hypertension. J. Exp. Med.

[b14] Devine A, Criddle RA, Dick IM, Kerr DA, Prince RL (1995). A longitudinal study of the effect of sodium and calcium intakes on regional bone density in postmenopausal women. Am. J. Clin. Nutr.

[b15] Fernandez R, Piechnik J, Fabris R, Malnic G, Fernandes LC (2004). Effect of chronic fish oil supplementation on renal function of normal and cachectic rats. Braz. J. Med. Biol. Res.

[b16] Fiore MC, Jimenez PM, Cremonezzi D, Juncos LI, García NH (2011). Statins reverse renal inflammation and endothelial dysfunction induced by chronic high salt intake. Am. J. Physiol. Renal Physiol.

[b17] Fischer R, Dechend R, Qadri F, Markovic M, Feldt S, Herse F (2008). Dietary n-3 polyunsaturated fatty acids and direct renin inhibition improve electrical remodeling in a model of high human renin hypertension. Hypertension.

[b18] Gold E, Gouldin A (1995). High dietary salt intakes lower bone mineral density in ovariectomised rats: a dual x-ray absorptiometry study. Bone.

[b19] Griel AE, Kris-Etherton PM, Hilpert KF, Zhao G, West SG, Corwin RL (2007). An increase in dietary n-3 fatty acids decreases a marker of bone resorption in humans. Nutr. J.

[b20] Hallfrisch J, Lazar F, Jorgensen C, Reiser S (1979). Insulin and glucose responses in rats fed sucrose or starch. Am. J. Clin. Nutr.

[b21] Heaney RP, Carey R, Harkness L (2005). Roles of vitamin D, n-3 polyunsaturated fatty acid, and soy isoflavones in bone health. J. Am. Diet. Assoc.

[b22] Hohenstein B, Hug CPH, Hausknecht B, Boehmer KP, Riess RH, Schmieder RE (2008). Analysis of NO-synthase expression and clinical risk factors in human diabetic nephropathy. Nephrol. Dial. Transplant.

[b23] Jones G, Beard T, Parameswaran V, Greenaway T, von Witt R (1997). A population-based study of the relationship between salt intake, bone resorption and bone mass. Eur. J. Clin. Nutr.

[b24] Kanbay M, Chen Y, Solak Y, Sanders PW (2011). Mechanisms and consequences of salt sensitivity and dietary salt intake. Curr. Opin. Nephrol. Hypertens.

[b25] Lin PH, Ginty F, Appel LJ, Aickin M, Bohannon A, Garnero P (2003). The DASH diet and sodium reduction improve markers of bone turnover and calcium metabolism in adults. J. Nutr.

[b26] Liu D, Veit HP, Wilson JH, Denbow DM (2003). Long-term supplementation of various dietary lipids alters bone mineral content, mechanical properties and histological characteristics of Japanese quail. Poult. Sci.

[b27] Lloberas N, Cruzado JM, Torras J, Herrero-Fresneda I, Riera M, Merlos M (2001). Protective effect of UR-12670 on chronic nephropathy induced by warm ischaemia in ageing uninephrectomized rats. Nephrol. Dial. Transplant.

[b28] Majid DSA, Williams A, Naver LG (1993). Inhibition of nitric oxide synthesis attenuates pressure-induced natriuretic responses in anesthetized dogs. Am. J. Physiol.

[b29] Massey LK, Whiting SJ (1996). Dietary salt, urinary calcium and bone loss. J. Bone Miner. Res.

[b30] Matavelli LC, Zhou X, Varagic J, Susic D, Frohlich ED (2007). Salt loading produces severe renal hemodynamic dysfunction independent of arterial pressure in spontaneously hypertensive rats. Am. J. Physiol.

[b31] Medeiros FJ, Mothe CG, Aguila MB, Mandarim-de-Lacerda CA (2005). Long-term intake of edible oils benefits blood pressure and myocardial structure in spontaneously hypertensive rat (SHR) and streptozotocin diabetic shr. Prostaglandins Other Lipid Mediat.

[b32] Mimran A, du Cailar G (2008). Dietary sodium: the dark horse amongst cardiovascular and renal risk factors. Nephrol. Dial. Transplant.

[b33] Nakayama T, Sato W, Kosugi T, Zhang L, Campbell-Thompson M, Yoshimura A (2009). Endothelial injury due to eNOS deficiency accelerates the progression of chronic renal disease in the mouse. Am. J. Physiol. Renal Physiol.

[b34] Newaz M, Blanton A, Fidelis P, Oyekan A (2005). NAD(P)H oxidase/nitric oxide interactions in peroxisome proliferator activated receptor (PPAR)alpha-mediated cardiovascular effects. Mutat. Res.

[b35] Sarić M, Piasek M (2005). Effects of sodium chloride on bone health. Arh. Hig. Rada Toksikol.

[b36] Shen CL, Yeh JK, Rasty J, Li Y, Watkins BA (2006). Protective effect of dietary long-chain n-3 polyunsaturated fatty acids on bone loss in gonad-intact middle-aged male rats. Br. J. Nutr.

[b37] Stoos BA, Carretero OA, Farhy RD, Scicli G, Garvin JL (1992). Endothelium derived relaxing factor inhibits transport and increases cGMP content in cultured mouse cortical collecting duct cells. J. Clin. Invest.

[b38] Sun D, Krishnan A, Zaman K, Lawrence R, Bhattacharya A, Fernandes G (2003). Dietary n-3 fatty acids decrease osteoclastogenesis and loss of bone mass in ovariectomized mice. J. Bone Miner. Res.

[b39] Tartibian B, Hajizadeh BM, Abbasi A (2010). The calciotropic hormone response to omega-3 supplementation during longterm weight-bearing exercise training in post menopausal women. J. Sports Sci. Med.

[b40] Terano T (2001). Effect of omega 3 polyunsaturated fatty acid ingestion on bone metabolism and osteoporosis. World Rev. Nutr. Diet.

[b41] Vargas F, Moreno JM, Rodríguez-Gómez I, Wangensteen R, Osuna A, Alvarez-Guerra M (2006). Vascular and renal function in experimental thyroid disorders. Eur. J. Endocrinol.

[b42] Watkins BA, Li Y, Lippman HE, Feng S (2003). Modulatory effect of omega-3 polyunsaturated fatty acids on osteoblast function and bone metabolism. Prostaglandins Leukot. Essent. Fatty Acids.

[b43] Ying WZ, Sanders PW (1999). Dietary salt increases endothelial nitric oxide synthase and TGF-β1 in rat aortic endothelium. Am. J. Physiol.

[b44] Yu CM, Burrell LM, Black MJ, Wu LL, Dilley RJ, Cooper ME (1988). Salt induces myocardial and renal fibrosis in normotensive and hypertensive rats. Circulation.

[b45] Zhang M, Hagiwara S, Matsumoto M, Gu L, Tanimoto M, Nakamura S (2006). Effects of eicosapentaenoic acid on the early stage of type 2 diabetic nephropathy in KKA(y)/Ta mice: involvement of anti-inflammation and antioxidative stress. Metabolism.

